# Data from the National Educational Panel Study (NEPS) in Germany: Starting Cohort 8 – Education for the World of Tomorrow in Grade 5 and Higher

**DOI:** 10.5334/jopd.179

**Published:** 2026-10-28

**Authors:** Lena Nusser, Katharina Molitor, Kathrin Lockl, Jana Kähler, Christoph Jindra, Timo Gnambs

**Affiliations:** 1 Leibniz Institute for Educational Trajectories, Germany; 2 Center for Research on Education and School Development (IFS), TU Dortmund University, Germany; 3 Leibniz Institute for Science and Mathematics Education, Germany; 4 Institute for Educational Quality Improvement, Germany

**Keywords:** Longitudinal, educational standards, secondary school, digital competence, civic literacy

## Abstract

The National Educational Panel Study (NEPS) is a longitudinal, multi-cohort study. Its most recent cohort, Starting Cohort 8, focuses on pathways through lower secondary school. Its initial sample, drawn in 2022, includes 6,141 students in fifth grade attending regular or special schools. Parents, school heads and teachers were also invited to participate in the survey. The longitudinal design, including annual competence testing and questionnaires provides research opportunities on competence development, its antecedents and consequences, through secondary school. A special feature is its connection to the *IQB Trends in Student Achievement*, a nationwide educational assessment in Germany, and new competence domains.

## (1) Background

The National Educational Panel Study (NEPS) is a large-scale longitudinal research program designed to collect representative data in Germany across different cohorts and age ranges (Blossfeld & Roßbach, 2019) hosted by the Leibniz Institute for Educational Trajectories (LIfBi). The NEPS is supported and implemented in cooperation with a nationwide network of researchers and institutions that make substantial contributions to the study design and the selection of content. Core focus of the NEPS is the development of competencies, processes of educational decision-making, learning environments, and the returns to education across the life course (Artelt & Sixt, 2023). “Starting Cohort 8 – Education for the World of Tomorrow” (NEPS-SC8) is the most recent of the total of seven NEPS starting cohorts so far.^1^ NEPS-SC8 follows students from the beginning of lower secondary education (Grade 5) through their transition into upper secondary education or vocational training. This paper addresses the rationale of NEPS-SC8, including its specific features and introduces the core contents of the first five waves.

Secondary school represents a critical phase in students’ educational biographies. Following elementary education, students are typically tracked into two or three different school types, largely based on their prior academic performance and grades (Matthewes, 2021). During this period, the foundations for subsequent educational pathways are established, often strongly shaping the range of educational opportunities and decision options available later in life. In addition, students undergo important developmental processes that may help and support them to gradually grow into responsible citizens (Harter, 2012; Sawyer et al., 2018). Adolescence is characterized by profound physical, emotional, and cognitive changes, which may pose challenges for students and can significantly affect academic achievement, the development of competencies (Branje et al., 2021) as well as students’ subjective well-being (Hoferichter et al., 2021).

Against this background, a central aim of NEPS-SC8 is to follow students’ development including competence development throughout and beyond this important phase. To incorporate information on relevant contexts and learning environments, their teachers, school heads and parents or legal guardians are also invited to participate in the study. NEPS-SC8 as the new school cohort was established twelve years after the launch of the NEPS Starting Cohort 3 – Grade 5 (NEPS-SC3; Thums et al., 2023) addressing students in secondary school.

A guiding principle in designing NEPS-SC8 was allowing for comparability with the existing NEPS-SC3 cohort to capture the effects of social and educational policy changes over the past decade. This includes comparable competence measures over time as well as the repetition of selected measures of survey instruments. Besides these aspects of comparability, the use of innovative survey methods (online surveys, computer-based administration within the classroom) and new content priorities were implemented. In addition, the survey program of NEPS-SC8 aims to reflect societal developments by incorporating new domains of competencies.

In recent years, digitalization and the digital culture have profoundly transformed the field of education. Digital media and tools are becoming increasingly important both in the classroom and at the school level (Drossel et al., 2020). Accordingly, a key educational objective is to enable students to use digital media and artificial intelligence systems confidently and competently. Therefore, for NEPS-SC8, the competence portfolio has been expanded to include a longitudinal assessment of digital competencies in addition to information and communication technology (ICT) literacy (Artelt, 2025). In addition, the use of digital media within and outside the school is also part of the survey program.

Likewise, growing societal complexity and political challenges have underscored the importance of civic literacy in education in recent years. Schools play a crucial role in preparing students to participate actively and responsibly in democratic societies (Alscher et al., 2022a). Accordingly, a key educational objective is to foster students’ understanding of civic institutions, political processes, and social participation, as well as their ability to critically reflect on societal issues. In response to these developments, for NEPS-SC8 the framework has expanded to include a longitudinal assessment of civic literacy. Additionally, topics related to civic engagement and democratic values are increasingly integrated into the survey program (Alscher et al., 2022b).

Moreover, NEPS-SC8 is linked to the *IQB Trends in Student Achievement* surveys (for example, Busse et al., 2022), a nationwide educational assessment in Germany, in close cooperation with the Institute for Quality Development in Education (IQB). Within the framework of the educational monitoring strategy of the Standing Conference of the Ministers of Education and Cultural Affairs (KMK), binding national educational standards define the subject-specific competencies students are expected to acquire at defined stages of schooling. Attainment of these standards is regularly assessed through the *IQB Trends in Student Achievement* studies, which are conducted at defined intervals in Grades 4 and 9 (e.g., Stanat et al., 2023; Stanat et al., 2025). In Grade 5, NEPS-SC8 administered tests developed by the IQB to assess educational standards in *German Reading* and *Mathematics* (Stanat et al., 2022). In addition, standards-based assessments will be repeated in Grade 9 for the same domains. The cooperation further enables the construction of a school panel by ensuring that a subsample of schools participating in NEPS-SC8 also took part in the *IQB Trends in Student Achievement 2022* (Stanat et al., 2023). In combination with school-level aggregated data from the IQB survey, this will enable the establishment of a panel of schools participating with Grade 9 classes in the IQB-survey 2022 and NEPS-SC8, allowing the investigation of school development questions.

This paper addresses the first five waves of NEPS-SC8 which will be continued beyond that timepoint.

## (2) Methods

### 2.1 Study design

The study has a longitudinal design and employs a mixed-mode survey approach. NEPS-SC8 was launched in 2022 and sampled students attending Grade 5 via sampled schools offering Grade 5 in all federal states of Germany. Data collection generally takes place once per year. In addition to students, teachers, school heads, and parents or legal guardians are invited to participate. In the event of students leaving the sampled school or schools withdrawing their consent to participate, these students will be tracked individually.

Student assessments including competence tests take place at school for one morning session and are administered by two trained interviewers using standardized procedures. The assessments are computer-based and conducted on standardized study laptops provided by the survey institute, ensuring identical hardware and testing conditions for the participants. A digital dashboard provides standardized instructions, enables the interviewers to unlock specific instruments, and allows them to monitor testing progress and timing. An exception applies to the standards-based tests in Grades 5 and 9, which are administered in a paper-and-pencil format (PAPI). Students who are still attending a participating school but are unable to participate in the school-based assessment are invited to complete an online questionnaire using their own devices at home. These questionnaires include the same questions as those asked in the school setting. However, no competence tests are administered.

Teachers and school heads, who can provide information on the school context and key characteristics of the learning environment, are surveyed every other year (Waves 1, 3, and 5). A novel feature of NEPS-SC8 is that all teachers at participating schools are invited to take part in the study. As a result, school-level information can be aggregated more comprehensively, providing not only selected attitudes and teacher perspectives but also a more detailed picture of the institution. Teachers and school heads receive an invitation link to an online questionnaire. Additional and specific survey contents are administered based on self-reported teaching roles (e.g., German teacher of the participating class). If questionnaires addressed to school heads remain unanswered, the school and its head are re-invited in the subsequent year to provide core school-level information via a shortened survey (Waves 2 and 4). In Waves 2 and 4, homeroom teachers are additionally asked to provide individual-level reports on their students.

Parents or legal guardians are surveyed annually. In Waves 1 and 3, they participate in computer-assisted telephone interviews (CATI) conducted by trained interviewers. In Waves 2 and from Wave 4 onwards, parents are invited to complete an online questionnaire.

Students who leave the initially sampled schools are surveyed individually. Beginning in Wave 2 (Grade 6), these students are contacted by telephone to maintain participation and to collect basic information about their current school situation. They are subsequently invited to complete a questionnaire, which can be answered either online or – if requested – paper-based. If telephone contact is unsuccessful, students receive an invitation to a slightly extended online questionnaire. Up to three reminders are sent to encourage participation, and with the second reminder, a paper-based questionnaire is additionally provided. Competence assessments for this group are conducted from Grade 9 onwards.

In general, data collection is conducted in cooperation with professional survey institutes. All online surveys are hosted by the coordinating institute.

### 2.2 Time of data collection

Data collection started in October 2022. Each year, school-based assessments as well as teachers and school heads interviews usually take place between October and January of the following year. An exception is Grade 9, where the time of data collection is aligned with the *IQB Trends in Student Achievement* survey, requiring an assessment at the end of the school year. Data from students who cannot participate in the school-based assessments are collected via online questionnaires after the school-based assessments are finished. Students, for whom individual tracking is implemented due to leaving the originally sampled school or because the school had withdrawn its consent to participate, are interviewed between October and May of each school year in order to include students identified as school leavers after the time frame for the school-based assessments. The survey period for parents runs from January to May (for telephone-based interviews) or April (for web-based interviews). An overview of the time periods of data collection is presented in Figure 1.

**Figure 1:**
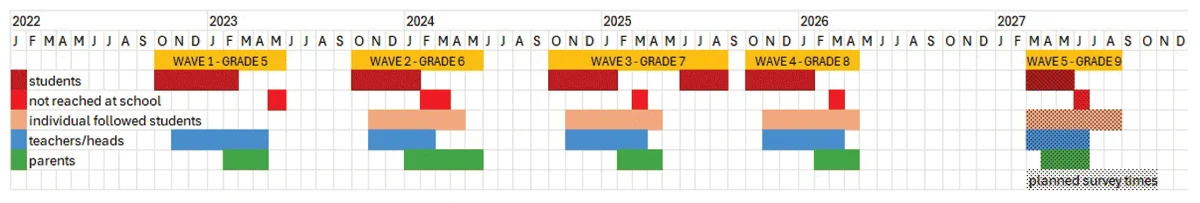
Overview of measurement points (Waves 1–5): students in school, individually follow-up students, teachers, heads of schools, and parents.

### 2.3 Location of data collection

Schools were sampled to be representative of German secondary schools in all 16 federal states starting in February 2022. To account for federal specificities, new schools were included in Wave 3. In Berlin and Brandenburg, elementary school has a duration of six years, requiring the addition of secondary schools in Wave 3. Moreover, some federal states (e.g., Hesse) provide the so-called orientation level wherein students continue to be taught together after Grade 4 for another two years. This time allows students, parents and teachers the opportunity for orientation, which secondary school type is best suited (Standing Conference of the Ministers of Education and Cultural Affairs, 2026). The school drop-out due to orientation levels after Grade 6 were compensated by sampling further secondary schools in Wave 3.

### 2.4 Sampling, sample and data collection

The target population of NEPS-SC8 is students in Germany attending Grade 5 in general education schools. This also includes special schools for all federal states and elementary schools for the federal states of Berlin and Brandenburg. The school sample was randomly drawn from a complete list of schools with replacements. Participation in the NEPS-SC8-study is voluntary at all levels. Schools without an academic track were oversampled to adequately represent the more diverse educational careers and trajectories of students attending these schools. Some of the recruited schools were participating in the *IQB Trends in Student Achievement 2022* (Stanat et al., 2023) allowing to build up a school panel. In schools that agreed to participate, all Grade 5 students and their parents were invited to take part in the study. In Wave 3, a school-level sample update was conducted to ensure that state-specific school conditions were also reflected in NEPS-SC8. Concurrently, students at schools and the corresponding grade level already participating in the study who had not yet provided consent to participate in NEPS-SC8 (e.g., because they were new to the school) were invited to participate. Further information on the sampling strategy can be found in the NEPS-SC8 data manual (FDZ-LIfBi, 2026).

Depending on state regulation, students receive a monetary incentive of 10 € for participation, a non-monetary incentive, or no incentive. Parents take part in a raffle – again depending on state regulations. Teachers and school heads receive non-monetary incentives.

The initial sample in Grade 5 consisted of *N* = 6,141 students whose parents or legal guardians gave consent for the child to participate. Of those, *n* = 5,472 took part in the school assessment or the online survey. Another *n* = 669 students could not be interviewed in school assessment and did not respond to the online survey either. About 3.5% of the sample attended elementary schools (*n* = 189) and 4% attended special schools (*n* = 213). Around half of the sample attended *Gymnasium* (*n* = 2,757; see Table 1). As participation in NEPS-SC8 is voluntary on school level as well as on an individual level, NEPS-SC8-data data do not constitute a fully representative sample of the target population. Compared to national administrative data for the school year 2022/2023 (Federal Statistical Office, 2023) it becomes evident that students attending *Gymnasium* are overrepresented in the sample (50.4% vs. 38.3%), while students attending comprehensive schools are underrepresented in NEPS-SC8-data (18.0% vs. 38.3%). In terms of, for example, lower secondary education (6.1% vs. 6.7%) and special schools (3.9% vs. 3.5%), the sample is close to the population in terms of distribution.

**Table 1:** School composition and student sample size of NEPS-SC8 in Grade 5 during autumn/winter 2022/2023.


SCHOOL TYPE	*n*	PERCENT

Lower secondary school (Hauptschule)	332	6.1

School with multiple tracks	558	10.2

Intermediate secondary school (Realschule)	437	8.0

Comprehensive school (Integrierte Gesamtschule)	986	18.0

Upper secondary school (Gymnasium)	2,757	50.4

Primary schools and orientation level	189	3.5

Special schools	213	3.9

Total	5,472	100


Students were on average 10.9 years old (*SD* = 0.8), and 50.4% of the sample were female. A small share (0.6%) did not specify a gender assignment or identified as diverse. Students’ socio-economic status according to the international socio-economic index of occupational status (International Socio-Economic Index of Occupational Status; Ganzeboom, 2010) reported by parents was on average 57.5 points (*SD* = 21.7) on a scale from 10 (indicating lower occupational status like subsistence farmers, fishers, hunters, and gatherers) to 90 (indicating higher occupational status like judge). Nearly a quarter (24.4%) had an immigrant background, defined as a student who was born abroad or had at least one parent who was born abroad. Parents (*n* = 4,597) gave their consent to participate in the NEPS-SC8, of whom n = 3,267 participated in the interview via CATI when their children were in Grade 5.^2^

As described in chapter 2.1 all teachers and school heads were invited to participate. In the first wave *n* = 2,496 teachers participated. A detailed overview of the roles the teachers had (homeroom teacher, math teacher, German teacher) can be found in the field reports (https://www.neps-data.de/Data-Center/Data-and-Documentation/SC8-Grade-5/Documentation).

### 2.5 Survey instruments

The study materials in NEPS-SC8 comprise different instruments, including competence tests across several domains as well as questionnaires for students and relevant context persons. Competencies constitute a central pillar of the NEPS framework. Given their unique role and central importance within NEPS, the domains of competence measurement of NEPS-SC8 are described in greater detail below. In addition, key thematic areas of the survey include learning environments, educational decisions, consequences of social inequality (e.g., migration), returns to education, and personality factors (Artelt & Sixt, 2023). Beyond these core areas, NEPS-SC8 also has a specific focus on current topics, namely education in a digital world, civic literacy and politics, well-being, institutional conditions, and further age-specific topics.

Due to the longitudinal design and the resulting large number of instruments and items implemented in NEPS-SC8, a comprehensive overview of all constructs, their measurement occasions, and the respective respondents is provided in tabular form in the appendix.

Detailed information on item wording and concepts assessed via questionnaires (not including competence assessments) can be found at https://www.neps-data.de/Data-Center/Data-and-Documentation/SC8-Grade-5.

A distinctive feature of the NEPS is the longitudinal assessment of educationally relevant competencies across secondary school years (Artelt et al., 2013; Autor*innenteam Kompetenzsäule, 2020). The competence assessments in NEPS-SC8 can be grouped into three broad categories: domain-general competencies, domain-specific competencies, and metacompetencies (Weinert et al., 2019). Domain-general competencies include cognitive basic skills (Lang et al., 2014), general knowledge (Schroeders et al., 2020), verbal and nonverbal reasoning (Heller & Perleth, 2000). Domain-specific competencies comprise reading competence (Gehrer et al., 2013), reading speed (Wolter & Gehrer, 2020), vocabulary at the word level (Lenhard et al., 2015), mathematical competence (Neumann et al., 2013), scientific literacy (Hahn et al., 2013), and native language competencies in Russian or Turkish (Schotte et al., 2026). Metacompetencies include ICT literacy (Senkbeil et al., 2013), digital competence (Artelt, 2025), and civic literacy (Alscher et al., 2022a). In addition, the NEPS-SC8 includes standards-based assessments of the *IQB Trends in Student Achievement* in reading (Bremerich-Vos et al., 2017) and mathematical competence (Reiss et al., 2017).

Compared to NEPS-SC3, the assessment in NEPS-SC8 places greater emphasis on education in a digital world. Therefore, the competence portfolio reflects this development and thus, NEPS-SC8 includes both ICT literacy and digital competence. Within the NEPS framework, ICT literacy encompasses technological and cognitive aspects of using digital tools. Digital competence complements these aspects by emphasizing competencies related to the critical reflection and the responsible handling of data (Artelt, 2025).

A second novelty is the emphasis on civic literacy. In addition to the questionnaires, a competence test is administered. The civic literacy framework covers knowledge items in terms of political structures, processes, and principles (Alscher et al., 2022b).

The competence tests are administered at different measurement occasions across waves and differ with regard to both administration mode and target population (see Appendix A1). While several assessments in Wave 1 (Grade 5), including reading competence, mathematical competence, verbal reasoning, and nonverbal reasoning, are administered as paper-based tests, most competence assessments in subsequent waves are implemented as computer-based tests. In addition, the *IQB Trends in Student Achievement* (of reading and mathematical competence are administered in paper-and-pencil format.

With regard to comparability with the existing NEPS-SC3 cohort, it is important to note that mathematics and reading competencies are assessed at the same measurement points (Grades 5, 7, and 9) and use the same test materials as in NEPS-SC3. Information on the comparability of the results between the two cohorts can be found in the respective technical reports (e.g., Gnambs, 2025, for reading competence and Petersen & Beyer, 2025, for mathematical competence in Grade 5). However, comparability is more limited for other competence domains because these domains were assessed at different grade levels and/or the test content was modified to reflect up-to-date requirements.

NEPS-SC8 also includes students attending special schools. To accommodate the specific learning conditions of students attending special schools, the assessment program is adapted with regard to test length, content, and administration procedures. Competence assessment focuses on two longitudinal assessments (i.e., reading and digital competence). In addition, domain-general competence assessments are complemented by specific tests on verbal and non-verbal reasoning (Gnambs & Nusser, 2024). Moreover, tests used for students with special educational needs feature instrument accommodations and adapted administration procedures for these student groups (Heydrich et al., 2013; Nusser et al., 2020). Accommodations include out-of-level-testing meaning that items originally developed for younger students are implemented in the survey (Gnambs & Nusser, 2024).

To enable longitudinal and cross-cohort comparisons of competence scores, the NEPS uses standardized linking procedures. Competence scores are numerical values that represent the level of competence estimated from a person’s responses to the test items. In the NEPS, these scores are estimated using weighted maximum likelihood estimates (WLEs; Warm, 1989). Because competence tests are adapted to the age and developmental level of the target population, identical test forms are generally not administered across waves. Linking refers to the statistical procedure used to connect scores from different test versions and measurement occasions, so that they can be interpreted on a common scale. This is achieved by using common information across assessments, most notably through anchor items that are administered in multiple test versions. As a result, competence scores from different waves and starting cohorts, including NEPS-SC8 and -SC3 (for reading or mathematical competence), can be directly compared and used to analyze developmental trajectories across the educational life course (Fischer et al., 2016).

To allow for valid population-based inferences and comparisons between NEPS-SC8 and NEPS-SC3, the data provide several weighting variables that account for unequal selection probabilities and nonresponse. Accordingly, two types of weights are available for each wave (Konrad et al., 2025; Konrad & Würbach, 2026). Cross-sectional weights refer to the target population of a given wave and should be used for research questions based on a single wave. In contrast, longitudinal weights refer to respondents who participated in multiple consecutive waves and are appropriate when analyzing changes over time within the same individuals.

Technical reports providing detailed information on the competence assessments and weights are continuously updated and made available on the NEPS website (https://www.lifbi.de/en-us/Start/Research/Publications/NEPS-Survey-Papers).

### 2.6 Quality Control

Data production in NEPS is the result of a comprehensive process to assure high data quality. The selection of the constructs to be included in the survey program is conducted by the NEPS network, an interdisciplinary commission of researchers and experts in the field. For NEPS-SC8, comparability with NEPS-SC3 played a central role, which led to the implementation of certain constructs across cohorts. At the same time, the selection process also took societal developments into account, resulting in new constructs that were specifically developed. The development of measurement instruments in NEPS is a standardized procedure including cognitive pretests, pilot studies and review processes within the German-wide network of expertise.

Unless data are collected via online surveys, they are collected by external survey institutes employing experienced interviewers. Interviewer training and training materials are developed jointly with these institutes. During field phases, data collection is continuously monitored by field visits, interim data deliveries, and structured feedback from the interviewers. Following data collection, item-non-response analyses are conducted and the survey programme for the following waves is adjusted if necessary.

### 2.7 Data anonymisation and ethical issues

All procedures conducted in this study complied with relevant national regulations, the principles of the Declaration of Helsinki, and applicable institutional guidelines. At the time of data collection, neither institutional nor national regulations required review or approval by a human-subjects research ethics committee. Research conducted within educational settings at German schools is subject to authorization by the respective federal states, which ensure compliance with established ethical standards and data protection regulations. NEPS-SC8 meets these legal and regulatory requirements and receives approval from the federal states of the participating schools, as well as from the responsible data protection authority at the coordinating institution for each wave. Participation in the study is entirely voluntary. The privacy and data protection rights of all participants are strictly safeguarded throughout the research process. Prior to data collection, informed consent is obtained from all participating teachers and the students’ parents or legal guardians. Once students turn 14 and 18 years, they are asked to provide informed consent to continue to participate in the study.

Data is anonymized to rule out reidentification. Three types of data are available which differ in their level of anonymization (see Section 3.7).

### 2.8 Existing use of data

The data has not yet been used in publications. However, previous publications with the NEPS in general have been reviewed in Gnambs et al. (2026) and are continuously documented on the NEPS website (https://www.neps-data.de).

## (3) Dataset description and access

### 3.1 Repository location

The most recent version of the data was published in 2026 (NEPS Network, 2026). With each new wave of data collection, the available scientific use files are updated accordingly. Information on the latest data version, including how to access the data, is available on the NEPS website: https://www.neps-data.de/Data-Center/Data-and-Documentation/SC8-Grade-5.

### 3.2 Object/file name

The data is provided in several files that can be linked using identifying variables beginning with “ID”. An overview of the 17 files is given in Table 2.

**Table 2:** Data files.


FILENAME	DESCRIPTION

SC8_CohortProfile	Metadata on all students in the sample to be used as starting point for all analyses

SC8_EditionBackups	Backup of original data that were modified during the data edition process

SC8_LinkTargetTeacher	Metadata for matching educators to students

SC8_ParentMethods	Metadata for the phone interviews with parents of the students

SC8_pCourseClass	Metadata and data from educators on their classes

SC8_pCourseGerman	Metadata and data from educators on their German classes

SC8_pCourseMath	Metadata and data from educators on their Math classes

SC8_pEducator	Data from educators

SC8_pInstitution*	Data from school heads

SC8_pParent	Data from parents

SC8_pTarget	Data from students (without competence assessments)

SC8_pTargetAssessments	Data from educators about individual students

SC8_TargetMethods	Metadata for student interviews

SC8_Weights	Generated sampling weights

SC8_xPlausibleValues	Generated plausible values for competence data

SC8_xTargetCompetencies	Test data for competence assessments in regular schools, including generated point estimates

SC8_xTargetSpecialNeedsCompetencies	Test data for competence assessments in special schools, including generated point estimates


*Note*. Plausible values are multiple imputed estimates of latent ability derived from item response models that reflect measurement uncertainty (Zink et al., 2026). * Only available as remote access (see Section 3.7).

The prefixes of the data files indicate panel data (p) in long format and competence data (x) in wide format. Data files without a specific prefix are generated by the NEPS Research Data Center to provide additional metadata.

### 3.3 Data type

The NEPS dataset consists of primary data collected longitudinally. In addition to the raw data, processed variables are provided, such as sampling weights, competence scores or generated sociodemographic variables (i.e., International Socio-Economic Index of Occupational Status, International Standard Classification of Education).

### 3.4 Format names and versions

The data are available in Stata (.dta) and SPSS (.sav) formats. The accompanying documentation on the NEPS website is provided as web pages (html) or as downloadable PDF. Additional analysis code supporting common data preprocessing tasks are provided as Stata .do files.

### 3.5 Language

The NEPS data and documentation are provided in German and English.

### 3.6 License

Because the NEPS data includes sensitive information which might potentially allow reidentification of individual respondents, the NEPS data is only available through controlled access. While individual researchers can use the data free of charge, access requires a valid data use agreement with the LIfBi. This agreement defines the conditions under which the data may be used and is limited to academic research and non-commercial purposes.

Detailed information on how to apply for a data use agreement is available on the NEPS website: https://www.neps-data.de/Data-Center/Data-Access/Data-Use-Agreements.

### 3.7 Limits to sharing

After signing the data use agreement, researchers have access to three types of data which differ in their level of anonymization and the availability of sensitive information:

- Download: The scientific use files have the highest level of anonymization and can be downloaded to the local computer. Sensitive information (e.g., detailed geographical data or exact birth dates) is removed or recoded to minimize the risk of disclosure.
- Remote access: A secure server environment provided by the LIfBi enables access via a virtual desktop over the internet and does not require local software installation. Following login and biometric authentication, more detailed information is available compared to the download version of the scientific use file.
- On-site access: Highly detailed microdata with the lowest level of anonymization (e.g., type of special educational needs) can be accessed at dedicated secure computer labs at the LIfBi.

As part of the funding agreement for the NEPS, analyses involving comparisons between German federal states are not permitted.

### 3.8 Publication date

The most recent version of the data was published in 2026 (NEPS Network, 2026). Previous data versions can be accessed via the NEPS website after login. Information on the release schedule for upcoming waves is available at https://www.neps-data.de/Data-Center/Overview-and-Assistance/Zeitplan-en-US.

### 3.9 FAIR data/Codebook

Each data version is uniquely identified by a DOI and can be accessed as described in Section 3.6. The datasets are available in multiple formats (Section 3.4) and can be processed using open-source software such as R (R Core Team, 2026). Extensive metadata accompany the datasets, including an online variable search that displays instruments, constructs, and the original survey questions. Comprehensive documentation, including data collection procedures, anonymization methods, and field reports, is freely available on the NEPS website.

To support effective use of NEPS data, the NEPS Research Data Center offers assistance via email and an online discussion board (*Forum4MICA*; https://forum.lifbi.de). It also provides thematic training courses held several times per year. Additional resources include the NEPS Survey Papers, which address methodological issues relevant to the use of NEPS data. Moreover, the R package *NEPSscaling* (Scharl & Zink, 2022; Zink et al., 2026) allows estimating plausible values for competence data in the NEPS.

## (4) Reuse potential

As described in the overview of the survey program, numerous constructs are assessed within NEPS-SC8 using a mixed-mode survey approach that incorporates the perspectives of students, teachers, school heads, and parents. Importantly, most constructs are assessed repeatedly, enabling the investigation of long-term developmental trajectories and their influencing factors within a longitudinal framework. With each wave, the research potential of the data expands and grows. Beyond the research topics that could already be addressed using data from NEPS-SC3 (Thums et al., 2023), NEPS-SC8 introduces several new focal areas that may be of considerable interest to researchers.

One focus area addresses the topic of education in a digital world and the question of how digital competencies can be successfully acquired. By capturing different perspectives, explanatory factors can be considered at two distinct levels: the individual characteristics of students, as well as those of the school and teachers. For example, individual factors such as affective-motivational prerequisites (e.g., different usage motives; Senkbeil, 2018) or cognitive prerequisites (e.g., reading competence) can be analyzed as influencing variables for successful acquisition. At the same time, school-related factors, such as the use of digital media in the classroom as well as teacher-specific characteristics, can be taken into account to assess the relative importance of these different factors. Furthermore, it is possible to investigate the extent to which the use of digital media has a compensatory effect or reinforces disparities when it comes to explaining social inequalities (see e.g., Drossel et al., 2020).

Moreover, data from NEPS-SC8 can be used to gain insights into the acquisition of civic literacy during this important phase of secondary education. In this context, it is possible to analyse the role of lesson characteristics (e.g., political content, open classroom climate) and teacher characteristics (e.g., understanding of politics) in fostering students’ understanding of civic institutions, political processes, and social participation, as well as their ability to critically reflect on societal issues. Furthermore, it allows for examining the extent to which the development of civic literacy is associated with other competencies, as well as with parents’ political interest and attitudes.

The inclusion of standard-based tests from the *IQB Trends in Student Achievement* studies in Grades 5 and 9 enables the analysis of the longitudinal relationship between this important element of the German educational monitoring system and other educational, motivational, and emotional outcomes. With the completion of the fieldwork for Grade 9, the previously mentioned school panel will also be completed, providing a unique opportunity to study changes over time using repeated data on average *German Reading* achievement for Grade 9 students within the same schools, as well as other student outcomes and inputs. This design addresses common issues in longitudinal surveys when school development questions are the focus, but students, rather than schools, are followed over time. To maximise research potential, NEPS-SC8 and the *IQB Trends in Student Achievement 2022* study (Stanat et al., 2023) collected additional information on selected constructs from the other survey, including teaching quality, school well-being, quality assurance and development measures, and school-based support programs. The collaboration, therefore, enables researchers, for example, to address questions such as: Are changes in classroom management over five years predictive of changes in school-level reading achievements among Grade 9 students?

A further potential for data reuse arises from the fact that all teachers within a school were invited to participate in the survey. As a result, comprehensive information on the school context and key characteristics of the learning environment is available, exceeding the information typically found in many other studies. Moreover, it allows to track developments at the school level based on a significant proportion of teachers per school.

Another advantage of using NEPS-SC8 data lies in its comparability with the existing NEPS-SC3 cohort, which enables the investigation of potential effects of social and educational policy changes over the past decade. Competence measures in the domains of mathematics and reading can be directly compared, as identical test materials were administered at the same measurement points in both cohorts. Although the modes of administration differed to some extent (paper-based in NEPS-SC3; predominantly computer-based in NEPS-SC8), linking procedures ensure that competence scores can be interpreted on a common scale.

However, some limitations should be acknowledged. First, comparisons between NEPS-SC8 and -SC3 are feasible for mathematical and reading competencies but not for other competence domains. This is because the grades at which these competencies were assessed were different, and/or the test content was adapted to reflect up-to-date requirements. Moreover, any comparison across cohorts must take into account potential differences in sample composition, partly because participation at both the school and student levels is voluntary. Accordingly, information on sampling procedures and sample weights should be taken into account (Konrad et al., 2025; Konrad & Würbach, 2026).

In addition, due to time constraints, a trade-off is inevitable between the number of constructs included in the survey program and the depth with which each construct can be measured (see also Thums et al., 2023). As a result, some constructs could not be assessed as frequently or as comprehensively as desired but were instead captured using a limited number of items.

Despite these limitations, the data offer substantial opportunities for analyzing the factors shaping the educational trajectories of secondary school students in Germany, while also incorporating new topics that may be of particular relevance in the current educational context.

## Additional File

The additional file for this article can be found as follows:

10.5334/jopd.179.s1 Appendix. Overview of NEPS SC8 survey program.

## Footnotes

Starting Cohort 7 (newborns) is scheduled to start at a later time point.

At the time of this contribution (September 2026) only data on the first two waves (Grade 5 and Grade 6) are available. We focus on the initial sample and therefore provided sample information on the first wave (Grade 5).
