# Supplementary material for: Data from the National Educational Panel Study (NEPS) in Germany: Starting Cohort 8 – Education for the World of Tomorrow in Grade 5 and Higher
Source: J Open Psychol Data. 2026 Oct 28;14:7. doi: 10.5334/jopd.179 (PMC13722009; doi:10.5334/jopd.179)
Supplement: Supplementary file 1 [file jopd-14-179-pr1.pdf]

Reviewer 1:

I assume that because this paper passed initial editorial checks, that it is OK. What I mean by "OK" is that it does not violate some ethical or legal guidelines. The NEPS is a massive undertaking, I am skeptical who is claiming credit for it here? Those who initially obtained funding (Blossfeld, etc.) are not co-authors. Why are these six authors claiming credit? Again, not really my task, but it does seem odd. For example, why is there nothing in the Funding Statement? The NEPS is heavily funded.

Thank you for raising this important issue. Large-scale assessment studies such as the NEPS rely on extensive collaborative efforts involving a broad network of researchers and staff who contribute to the study design, the selection of constructs, the development of instruments, and the implementation of the study across waves. We certainly do not intend to claim credit for the entirety of NEPS-SC8. Rather, the authors, who are affiliated with different institutions, were closely involved in specific aspects of the preparation and implementation of NEPS-SC8 and made substantial contributions to the present manuscript. We also explicitly acknowledge the broader NEPS consortium and its contributions in the acknowledgements section. In addition, we have strengthened this point in the Introduction to make the collaborative nature of the project even clearer.

I also would want to know from the authors at the beginning of the paper, why it is important to introduce this data as unique and uniquely useful to psychologists compared to all other waves of NEPS data. Otherwise, why not introduce the entire NEPS here? I understand that the authors have pointed to some of this in the Reuse Potential section, and I find this good, but it is not clear to me how much of this is unique to NEPS-SC8 (and what about future planned cohorts? As in: is this unique to NEPS-SC8 or has the NEPS permanently changed now for all future cohorts to use these new advances in survey mode and questions?).

Thank you for pointing out this lack of clarity. It is true that the NEPS aims to assess key constructs across all cohorts. Consequently, all starting cohorts are of considerable interest to psychological research. However, despite many similarities, the NEPS starting cohorts differ substantially in several aspects. These differences include, for example, the sampling design (institution-based versus individual-based sampling), the inclusion of different contextual respondents, and assessment schedules that are tailored to the developmental processes and transitions expected within specific age ranges. Thus, decisions for NEPS-SC8 are not necessarily also equivalent for future cohorts. The NEPS continuously evolves in response to methodological innovations and societal changes. As such, future developments may require adaptations in study design, instruments, and content to ensure the continued relevance of the data. Since not all future societal developments and research needs can be anticipated at present, the NEPS remains committed to continuously identifying and integrating new approaches and innovations.

Given these cohort-specific characteristics, we believe that a comprehensive presentation of the entire NEPS framework would be beyond the scope of the present manuscript. Moreover, several general introductions to the NEPS have already been published (e.g. Artelt & Sixt, 2023; Blossfeld et al., 2011; Blossfeld et al., 2019; Roßbach & von Maurice, 2018; von Maurice et al., 2016). Therefore, we believe that including a broader description of all starting cohorts would provide only limited additional value for readers. Rather, our goal was to provide a concise and coherent overview of recent developments within the NEPS and make the features of this new starting cohort accessible to a broader readership.

We have revised the manuscript to clarify the rationale for focusing on the NEPS Starting Cohort 8 more explicitly.

Artelt, C., & Sixt, M. (2023). The National Educational Panel Study (NEPS) - Framework, design, and research potential. *Zeitschrift für Erziehungswissenschaft, 26*(2), 277-298. https://doi.org/10.1007/s11618-023-01156-w
Blossfeld, H.-P., von Maurice, J., & Schneider, T. (2011). The National Educational Panel Study: Need, main features, and research potential. *Zeitschrift für Erziehungswissenschaft, 14*(2), 5–17. https://doi.org/10.1007/s11618-011-0178-3
Blossfeld, H.-P., Von Maurice, J., & Schneider, T. (2019). The National Educational Panel Study: Need, main features, and research potential. In H.-P. Blossfeld & H.-G. Roßbach (Eds.), *Education as a Lifelong Process* (pp. 1–16). Springer Fachmedien Wiesbaden. https://doi.org/10.1007/978-3-658-23162-0_1
Roßbach, H.-G., & von Maurice, J. (2018). Das Nationale Bildungspanel als wertvolle Ressource für die Bildungsforschung. In R. Tippelt, & B. Schmidt-Hertha (Hrsg.), *Handbuch Bildungsforschung* (pp. 739–756). Springer. https://doi.org/10.1007/978-3-531-19981-8_31
von Maurice, J., Blossfeld, H.-P., & Roßbach, H.-G. (2016). The National Educational Panel Study: Milestones of the years 2006 to 2015. In H.-P. Blossfeld, J. von Maurice, M. Bayer, & J. Skopek (Hrsg.), *Methodological issues of longitudinal surveys: The example of the National Educational Panel Study* (pp. 3–18). Springer. https://doi.org/10.1007/978-3-658-11994-2_1

In the Methods section they talk about 2022 and Grade 5, but then they proceed to discuss an exception in Grade 9. If I understand correctly, the panel has not yet reached Grade 9, so… what are the talking about? Or are they talking about other NEPS cohorts? This applies to the following paragraphs where it feels like they are describing the NEPS in general, nothing special to NEPS-SC8, which begs the question why we have a paper uniquely devoted to SC8 in theory, when it is more devoted to the NEPS in general when one reads it. I think this gets at a problem of tense in this paragraph and others, sometimes the sentences are phrased in the past tense, occasionally future tense and often present tense. Difficult to follow. This includes the Waves also. What is Wave 3? Every NEPS cohort gets a Wave 3 and they are talking generically about every NEPS cohort, or only NEPS-SC8?
Thank you again for pointing out this lack of clarity. As noted above, this paper focuses exclusively on NEPS Starting Cohort 8. While some features reflect the general design and scope of the NEPS, we have revised the manuscript to ensure a more consistent use of tenses and to further strengthen the focus on NEPS-SC8 throughout the text.
Planning for upcoming assessment waves, particularly in the school context, typically begins several years in advance. Therefore, although Wave 5 (Grade 9) has not yet entered the field, the study design and the planned content have already been specified and can be described.

They spend a lot of time discussing the IQB survey. I think it would be useful to have a background paragraph somewhere that talks about how this survey was started (like they do with the NEPS) and how the two surveys came to cooperate, and/or if this was planned from the beginning. Also, very important is whether this cooperation is only in NEPS-SC8 or in other cohorts?
Thank you for this comment. In response to a comment by reviewer #2, we added a sentence to the reuse potential describing the IQB Trends in Student Achievement studies as part of the German educational monitoring strategy of the Standing Conference of the

Ministers of Education and Cultural Affairs (KMK). We further clarified that this is the first time a NEPS starting cohort has been linked to the Trends in Student Achievement studies.

I would find it helpful to add a date range to Table 1 so that we can understand that this is a snapshot at this moment in time. I could also imagine that they offer the population of Germany composition in an additional column so that we can see how the NEPS distribution reflects the full census.
Thank you for this important comment. We added the information on the time range of data collection in the title of Table 1. NEPS-data aim to represent the heterogeneity of German school types within federal states and thus students' social heterogeneity (more information on the sampling strategy can be found in the NEPS-SC8 data manual (FDZ-LIfBi, 2026) NEPS data, however, do not constitute a representative sample of the target population, as participation of schools and students within schools is voluntary. We added additional information on the distribution of Grade-5-students to school types in Germany in the school year 2022/2023.

FDZ-LIfBi. (2026). Data Manual NEPS Starting Cohort 8– Grade 5 (2022). Education for the World of Tomorrow, Scientific Use File Version 2.0.0. Leibniz Institute for Educational Trajectories, National Educational Panel Study.

Regarding the last point, what about weights? The authors present the weight variable and offer sources to find out more information, but I think the reader would want to know right away if they can use the NEPS to have a census representative sample, or what kind of sample this is after weighting.
We appreciate the helpful comment. Therefore, we added a brief description on the weight variables provided in the data as part of the survey description (Section 2.5) to inform readers about the available information.

Minor points:
I find "gradually grow into responsible citizens" as inaccurate. I think that they want to state that the NEPS measures aspects citizenship and societal engagement? For sure many school children do not grow into "responsible adults" (think about crime rates, tax avoidance, not following ordinances, etc.).
Thank you for raising this aspect. We followed your recommendation and revised the sentence.

I believe that "Moreover, the survey is closely linked to the IQB Trends in Student Achievement surveys" paragraph describing the IQB and NEPS cooperation and school-level linked data paragraph is too long. I think they can make all their points in about half of the space.
We followed the recommendation and substantially shortened the paragraph.

Reviewer 2

Review of the manuscript "Data from the National Educational Panel Study (NEPS) in Germany: Starting Cohort 8 – Education for the World of Tomorrow in Grade 5 and Higher"

The manuscript describes the data and related documentation from NEPS Starting Cohort 8, which has been collecting data in annual waves since 2022. The objective of the panel study is to generate longitudinal empirical data that facilitate research on the challenges and opportunities of education in a changing world. The target population consists of students who attended Grade 5 in regular and special schools in Germany in 2022. During the data collection waves, students participated in interviews and competence tests. In addition, data were collected from their parents, teachers, and school principals. The NEPS-SC8 data constitute a valuable resource for researchers investigating the consequences of societal and educational policy developments on students and educational trajectories. This paper complements the 2023 JOPD publication on NEPS Starting Cohort 3, which also targeted students in Grade 5 at recruitment.

Overall, the manuscript is well written, informative, and follows the structure and style expected for a JOPD data paper. It provides a comprehensive overview of the study design, data collection, available instruments, and data access, making the dataset highly valuable for the research community. I have a few specific comments and suggestions, as well as some points regarding formal aspects of the manuscript, that I believe will further improve the clarity and usability of the manuscript. Overall, these are relatively minor issues that should be straightforward to address.

We would like to thank the reviewer for the appreciative and constructive comments. We sincerely appreciate the time and expertise you dedicated to helping us improve the quality and clarity of our work.

Specific comments

- Page 2: "Starting Cohort 8 – Education for the World of Tomorrow" (NEPS-SC8) is the most recent of the total of seven NEPS starting cohorts so far." For readers unfamiliar with the NEPS, the reference to Cohort 8 out of a total seven cohorts is not immediately intuitive. Consider adding a footnote explaining that Cohort 7 is scheduled to start in 2031.

Thank you for this hint. We added a footnote.

- Page 2: "This new school cohort was established twelve years after the launch of the NEPS Starting Cohort Grade 5 …" I suggest specifying the cohort number at this point, as the following paragraph refers only NEPS-SC3. On my first reading, I assumed that the authors mistakenly were referring to NEPS-SC5 in this sentence.

Thank you for pointing out this lack in clarity. In fact, SC3 is also referred to as the "Starting Cohort Grade 5." To avoid any ambiguity, we have now specified the cohort number.

- Page 2:" Besides these aspects of comparability, the use of innovative survey methods (online surveys, computer-based assessment within the classroom) …" Although the term "assessment" is used in the abstract, this is the first point in the main text where it is introduced. Consider clarifying that it refers to a cognitive test, as not all readers may be familiar with the terminology related to the measurement of competences.

We appreciate the thorough review of the manuscript. At this point, our intention is to emphasize the technologies used in NEPS-SC8 rather than the assessed content domains or cognitive tests. This aspect is elaborated on in the subsequent sentences. To better reflect this focus, we replaced "assessment" with "administration".

- Page 4: "… computer- or web-based instruments ..." These terms appear somewhat ambiguous, as web-based instruments are a specific type of computer-based instruments. Consider clarifying the terminology.
Thank you for this important note. We agree that the terminology was not sufficiently distinct. To improve clarity, we now refrain from using the general description but rather explicitly describe the different assessment modes in the following paragraphs with respect to each target group and subsample.

- Page 4: "Students who are unable to participate in the school-based assessment are invited to complete an online questionnaire." In the previous sentences, the authors describe the role and tasks of interviewers when assessments are conducted in the school setting. However, students who are absent during the school-based assessment complete the assessment online. Could the authors clarify whether these online assessments are also conducted in the school environment and supervised by interviewers, or whether they are completed at home? In both cases, are the same assessment standards maintained? How is the situation handled when students complete the assessment without interviewer support (e.g., for unlocking instruments)? Are the results from both modes of administration comparable, given that the test setting is a critical factor in the assessment of cognitive skills?
Thank you for pointing out the lack of clarity in this section. For those students, no competence tests were administered. We clarified this point in the paper.

- Page 5: "… interviews usually take place between October and January of each year, …" The wording is potentially misleading, as the interview period crosses the calendar year boundary.
Thank you, we followed your recommendation.

- Page 6: "… on a scale from 10 to 98." Consider clarifying that higher values indicate a higher socio-economic status, as this interpretation may not be familiar to all readers.
- Page 6/7: "N = 4,597 parents gave their consent to participate in the NEPS-SC8, of whom n = 3,267 participated in the interview via CATI (computer-assisted telephone interview) in Grade 5." The wording is potentially misleading, as "in Grade 5" appears to refer to the parents rather than the students. Consider clarifying that Grade 5 refers to the students' grade level at the time of data collection (e.g., Overall, N = 4,597 parents gave their consent to participate in NEPS-SC8. Of these, n = 3,267 participated in a CATI (computer-assisted telephone interview) when their children were in Grade 5).
Thank you for these notes. We followed your recommendation, included information on the coding and the occupational status and clarified the reference of "Grade 5"

- Footnote 1, page 7: "At the time of this contribution (spring 2026) …" Consider updating the date information, as the publication of this article may be expected no earlier than summer 2026 or even autumn 2026.
We have revised the footnote accordingly and specified the relevant date.

- Page 7: "A detailed overview of the roles … can be found in the field reports." Please provide a reference or link to the field reports.
Thank you, we followed your recommendation and added a link.

- Page 7: “Detailed information on item wording and concepts …” The sentence does not make it entirely clear what “item wording and concepts” refers to. Please clarify whether this applies to all instruments in general or to specific instruments.
We followed the recommendation and added the information that these information relate to questionnaires not competence assessments.

- Page 8: “Moreover, tests used for students with special educational needs feature instrument accommodations (e.g. out of level; …” The meaning of “out of level” is unclear. Please provide a brief clarification or definition.
We followed the recommendation and added an explanatory sentence about out-of-level testing.

- Page 8: “To enable longitudinal and cross-cohort comparisons of competence scores, the NEPS applies standardized linking procedures that place assessments administered at different measurement occasions on a common scale.” Readers who are not familiar with competence assessment studies may benefit from a brief explanation of terms and concepts such as test scores, linking, and scaling. Without this context, statements such as this one may be difficult to understand.
Thank you for this helpful comment. We agree that the original wording assumed familiarity with competence assessment and psychometric terminology. We have therefore revised the paragraph to provide a brief explanation of competence scores and statistical linking. Specifically, we now clarify that competence scores are numerical values representing a person’s estimated level of competence and that, in the NEPS, these scores are expressed as weighted maximum likelihood estimates (WLEs). We also explicitly explain that linking connects scores from different test versions and measurement occasions, allowing them to be interpreted on a common scale.

- Page 9: "Technical reports providing detailed information on the competence assessments and their administration are continuously updated and made available on the NEPS website.” Please insert a link to the section of the NEPS website where these technical reports are accessible.
Thank you, we followed your recommendation.

- Table 2, page 10/11: Consider briefly explaining “plausible values” in the table notes for readers who are not familiar with competence assessment studies.
We followed the advice and included a brief description of “plausible values” in the table notes.

- Page 11: “The prefixes of the data files indicate panel data (p) in long format, spell data (sp), and competence data (x) in wide format.” The data files listed in Table 2 do not include a spell data file. Please clarify whether this information should be removed or whether spell data will be included in upcoming, not yet published data files. If spell data files are planned for future releases, please briefly explain their purpose and how they differ from the other files (e.g., in terms of content and layout), as not all readers may be familiar with these data structures.
We appreciate the thorough reading of our manuscript. It is true that spell data is currently not part of the data. Therefore, we removed the respective information.

- Page 13: „The inclusion of standard-based tests in Grades 5 and 9 enables the analysis of the longitudinal relationship between this important element of the German educational monitoring and other educational, motivational, and emotional outcomes." I assume that by "German educational monitoring" you refer to the German concepts of Bildungsberichterstattung or Bildungsmonitoring. First, the English term may reflect the terminology used at LIfBi but it may not be readily understandable to readers who are unfamiliar with NEPS, IQB Trends in Student Achievement etc. Second, please briefly explain what is meant by these terms and how they relate to the standard-based tests described here.

Thank you for your comment. We added a sentence in the Background section that clarifies that we are referring to the German educational monitoring strategy of the Standing Conference of the Ministers of Education and Cultural Affairs (KMK). We further clarify that nationally binding educational standards and their periodical assessment are just one element of the overall strategy.

- Page 14: "First, comparisons between NEPS-SC8 and -SC3 are feasible for certain competencies and aspects of the survey program, but not for all." While this information is relevant, it would be helpful to provide more details. In particular, please clarify which competence domains are affected, the reasons why comparisons are not possible, and where users can find further information. It is also unclear whether the reference cited at the end of the paragraph also refers to this sentence.

Thank you for this helpful comment. We agree that the information provided in the original manuscript was too vague. We have therefore expanded Section 2.5 to clarify the comparability of the competence measures between NEPS-SC3 and NEPS-SC8. We have also clarified this issue in Section 4 ("Reuse Potential"). In addition, we hope it is now clearer that the references cited at the end of the paragraph pertain to information on sampling procedures and sample weights rather than to the comparability of the competence measures.

Formalities

- Spell out all acronyms (e.g., AI and ICT on page 3, ISCED on page 11) when they are introduced for the first time.

Thank you, we followed your recommendation.

- Page 2: In the reference list, the reference for Matthewes is listed as 2021. Although the article was published online in 2020, the journal's recommended citation gives the publication year as 2021.

Thank you, we followed your recommendation.

- Page 6: "n = 5,472 took part in the school assessment or the online survey. n = 669 students could not be interviewed in school assessment and did not respond to the online survey either." Consider integrating sample sizes into the running text rather than beginning sentences with n = ... (e.g., Altogether, 5,472 students took part …).

Thank you, we followed your recommendation.

- Page 6/7: "N = 4,597 parents gave their consent to participate in the NEPS-SC8, of whom n = 3,267 participated in the interview via CATI (computer-assisted telephone interview) in Grade 5." This sentence also starts with "N = …"

Thank you, we followed your recommendation.

- Page 6: “Approx. 3.5 % of the sample attended elementary schools (n = 189) and 4 % attended special schools (n = 213).” Avoid sentences to start with an abbreviation.

Thank you, we followed your recommendation.

- Table 1, page 6: The ordering of the school categories could be reconsidered, as it is not immediately intuitive. Furthermore, I suggest reporting percentages with one decimal place only, as reporting a second decimal place may imply a level of precision that is not warranted (this also applies to figures reported in the following text passage).

Thank you for pointing this out. We decided not to change the ordering of the categories, as it is aligned with the variables available in the Scientific Use Files. However, we have added further information on the different school types to improve clarity. In addition, we refrained from reporting values to a second decimal place.

- Page 6: The reference Ganzeboom, 2010 is missing in the reference list.

Thank you, we followed your recommendation.

- Page 8: Please clarify whether the Alscher et al. reference is 2022a or 2022b.

Thank you, we followed your recommendation.

- Page 8: “In addition, the NEPS-SC8 included standards-based assessments … mathematical (Reiss et al., 2017) competence.” Consider moving the Reiss et al. reference to the end of the sentence.

Thank you, we followed your recommendation.

- Page 13: “Furthermore, it is possible to investigate the extent (…) to explaining social inequalities (see e.g., Drosssel et al., 2020).” Note that the cited reference contains a typo.

Thank you, we followed your recommendation.

- Check the reference Steger & Schütz (2025), as it is listed in the reference list but does not appear to be cited in the text.

Thank you for your careful reading, we omitted the reference

- The reference Standing Conference of the Ministers of Education and Cultural Affairs (2025) is not correctly positioned in the reference list and should be moved after the references by Stanat et al. to follow the alphabetical order.

Thank you, we followed your recommendation.

Appendix

- Throughout all tables, there appears to be a typo in the Wave 2 column header: (2023/23) should read (2023/24).

Thank you, we corrected the typos.

- At first, it is not immediately clear that the superscript numbers refer to the references listed at the end of the appendix document. A reader would expect an explanation of these numbers in the table notes. In addition, the references will most likely be integrated into the

overall reference list of the article, in which case the numerical correspondence would be lost.
Thank you for raising this issue. Including the references from the appendix in the overall reference list does not seem to be a good solution, as the main text does not refer to most of these sources. Instead, we added a sentence to the explanatory notes clarifying the meaning of the superscript numbers (see below).

- At the beginning of the table (section Competences), each competence domain listed in the first column is followed by an abbreviation in parentheses. It can be assumed that these are the abbreviations for the respective competence domain, but this is not explicitly explained. It is also unclear why these abbreviations are needed or what purpose they serve. If the abbreviations are not essential, consider omitting them for simplicity.
We followed the recommendation and omitted the abbreviations.

- In the first section of the table (Competences), the letters "P" and "C" used within the table refer to the mode in which the assessment tasks were administered. Only the subscript "sen" refers to the respondent group, namely students at special schools. In the subsequent sections of the table, however, "S" is consistently used to denote students. This difference in notation may be confusing. Consider clarifying that, in the Competences section, "P" and "C" refer to the assessment mode for students in regular schools, while the subscript "sen" identifies the special-school student group.
Thank you for raising this point. We added an explanatory note at the beginning of the appendix.

- Spell out acronyms in the notes (e.g., AI, HR, ICT).
Thank you, we followed your recommendation.

- There appears to be a typo in the header: “Digital medien & use” should read “Digital media & use”.
Thank you, we followed your recommendation.

- The abbreviation “kindl” in parentheses after “Health status” may not be familiar to all readers. Consider briefly explaining that KINDL is the name of the instrument used to assess health-related quality of life in children and adolescents.
Thank you, we followed your recommendation.

- In several instances (e.g., for the item “History of education” and several items in the section “Institution”), the symbol “H” for the respondent group “head of school” is listed in parentheses. Is this intentional? If so, the parentheses’ meaning is not clear to readers
Thank you for drawing our attention to this issue. We have added an explanation of the symbol (H).
