# Supplementary material for: Data from the National Educational Panel Study (NEPS) in Germany: Starting Cohort 8 – Education for the World of Tomorrow in Grade 5 and Higher
Source: J Open Psychol Data. 2026 Oct 28;14:7. doi: 10.5334/jopd.179 (PMC13722009; doi:10.5334/jopd.179)
Supplement: Supplementary file 2 [file jopd-14-179-s1.pdf]

Appendix.

Overview of NEPS SC8 survey program.

This appendix provides an overview of the survey content, its measurement points, and the informant groups in NEPS-SC8.

For the competence assessments, the participating students are always the respondents. The letters P (paper-based) and C (computer-based) refer to the assessment mode. The subscript "sen" indicates students attending special schools.

For all other questionnaire content, the letters indicate the respondent group: S = student (target person), P = parent, T = teacher, and H = head of school, (H) = head of school who did not respond in the previous wave.

Superscript numbers refer to references listed at the end of the appendix.

Competences [1]	Wave 1 (2022/23)	Wave 2 (2023/24)	Wave 3 (2024/25)	Wave 4 (2025/26)	Wave 5 (2026/27)
**Domain-general competences**					
Reasoning			C, C_{sen}		
General knowledge		C, C_{sen}			
Verbal reasoning [2]	P_{sen}			C_{sen}	
Nonverbal reasoning [2]	P_{sen}			C_{sen}	
**Domain-specific competences**					
Reading competence* [3]	P, P_{sen}		C, C_{sen}		C, C_{sen}
Reading competence (IQB Trends in Student Achievement) [4]	P				P
Reading speed [5]			C, C_{sen}		
Receptive vocabulary		C, C_{sen}			
Mathematical competence* [6]	P		C		C
Mathematical competence (IQB Trends in Student Achievement)	P				P
Scientific competence* [7]				C	
Languages of origin Russian and Turkish		C			
**Meta-competences**					
Information and communication technology (ICT)* [8]		C		C	
Digital competence		C, C_{sen}		C, C_{sen}	
Civic literacy [9]			C		

P = Paper-based Test, C = Computer-Based Test, P_{sen}/C_{sen} = administrated to students at special schools. *Competence scores are linked over time.

Learning environments [10]	Wave 1 (2022/23)	Wave 2 (2023/24)	Wave 3 (2024/25)	Wave 4 (2025/26)	Wave 5 (2026/27)
**Learning opportunities in formal learning environments**					
Instructional quality [11]	S, T		S, T		S, T
**Family/home learning environment**					
Home-based- parental involvement in education (quantity)	S	S	S	S	S
School-based parental involvement in education (quantity)			P	P	P
School based parental involvement (quality)	S		S, P	P	S, P
Parental availability		S		S	
**Learning opportunities in nonformal/informal learning environments**					
Regular activities (cultural activities) [12]	S	S	S	S	S
Privat tutoring	S	S	S	S	S
**School development**					
Human ressources development: importance of further professional training	T		T		T
Leadership	T		T		T
Teacher evaluation/feedback	T		T		T
Stakeholder participation	T		T		T
Satisfaction with autonomy in the workplace	T		T		T
School innovativeness culture	T		T		T

School and instructional development	T		T		T
School life: dealing with diversity; openness towards diversity	T		T		T
Self-efficacy (teaching, classroom management, student motivation)[11]	T		T		T
Teacher cooperation	T		T		T
Transactional leadership	T		T		T
**Digital media & use**					
Use of digital media in schools	S, T	S	S, T	S	S, T
School culture: Attitudes toward school and the internet	S	S	S	S	S
Digital media in the home		S		S	
ICT-related reasons for use		S		S	
ICT-related educational practices		S, P		S, P	
Digital games		S		S	
Academic Media-Multitasking			S		S
Technoference				S, P	
Use of artificial intelligence				S	
Parental self-assessed digital media competence		P		P	
Teaching quality: Use of digital media	T		T		T
Teaching AI in the classroom					T
Educational concept: Digital media	T		T		T

Teachers' self-efficacy in computer tasks	T	T	T
Teachers' emphasis on ICT in teaching	T	T	T
Teachers' views on using ICT for teaching and learning at school	T	T	T

Note. S = student (target person), P = parent, T = teacher, H = head of school.

Educational decisions [13]	Wave 1 (2022/23)	Wave 2 (2023/24)	Wave 3 (2024/25)	Wave 4 (2025/26)	Wave 5 (2026/27)
**Bounded rationality/norms/framing**					
Status maintenance	S	P	S	P	S, P
Aspirations	S	S, P	S, P	S, P	
Generalized attitude towards education			S	P	
Preferences / career orientations / meaning of work					S
**Cultural capital**					
Objectified cultural capital	S				S, P
Reading (quantity)	S	S, P	S	S	S, P
Number of books read	S	P	S	P	S, P
Participation in high culture and popular culture	S	S, P	S	S	S, P
Parental support for activities				P	P
**Social capital**					
Normative influence (reference groups)	S		S		S
Social capital within the family		S	P	S	P
Resource transmission (support, information, obligations) – education/career					S
Majority of friendships		S	S		
Position generator [14]		P			P
Social composition of the school	H, T	H	H, T	H	H, T
Student-specific assessments (german and		T		T	

mathematics competencies)			
Stereotyped assessment of reading and mathematics competencies	T	T	T

Note. S = student (target person), P = parent, T = teacher, H = head of school.

Migration [15]	Wave 1 (2022/23)	Wave 2 (2023/24)	Wave 3 (2024/25)	Wave 4 (2025/26)	Wave 5 (2026/27)
**Cultural orientation**					
Integration aspects (language; german identity, friends)	S	S	S, P	S, P	S, P
Identity - acculturation orientation - country of origin	S		S		S, P
**Language**					
Language(s) of origin	S, T		T		T
Language use		S		S	
**Migration-specific learning environment**					
Attitudes towards multilingualism (in the classroom)			T	S	T
Approaches to multilingualism in the classroom	T		T		T
Ethnic self-identification / perceived ascription by others			S, T		S
Share of students/teachers with migration background within class /school	H, T	H	H, T	H	H, T
General factors of educational success			P		P
**Perceived discrimination**					
Perceived discrimination (group-specific and personal) [16]			S, P		S, P

Note. S = student (target person), P = parent, T = teacher, H = head of school.

Returns to education [17,18]	Wave 1 (2022/23)	Wave 2 (2023/24)	Wave 3 (2024/25)	Wave 4 (2025/26)	Wave 5 (2026/27)
**Satisfaction**					
Life satisfaction	S	S	S, P	S, P	S, P
Satisfaction with life domains		S		S	S
**Political participation**					
Political interest	P		S	S, P	S
Actual participation in political actions			S	S	
Understanding of democracy			S		
Youth-specific political items (discussions / media)			S		
Political deprivation			S	S, P	
Conspiracy mentality			S	S, P	
Attitudes towards migration				S	P
**Social participation** [19]					
Social trust			S		
Social belonging				P	P
Social exclusion	S	S	S	S	S
Memberships		S			S, P
**Health** [20]					
Health behavior				S, P	P
Health status (KINDL-instrument assessing health-related quality of life in children and adolescents)		S		S	

Self-rated health	S	S	S	S, P	S, P
Height / weight		S		S	
Parents’ subjective assessment of health				P	P
Preferences (time and risk)					S, P

Note. S = student (target person), P = parent, T = teacher, H = head of school.

Motivation & personality [21]	Wave 1 (2022/23)	Wave 2 (2023/24)	Wave 3 (2024/25)	Wave 4 (2025/26)	Wave 5 (2026/27)
**Self-concept**					
Self-concept school [22]	S	S, T	S	S, T	S
Self-esteem			P	P	S, P
**Motivation**					
Intrinsic motivation	S	S	S	S	S
**Coping with everyday school life**					
School anxiety		S		S	
Career interests					S
**Personality**					
Ambiguity tolerance[23]				S	
Big five			S		
Self-control			S		
Emotion regulation					S
Trust in science [24]				S	
Gender roles [25]		S	P	S	P
Behavioural strengths and difficulties [26]		S	P	S	S, P

Note. S = student (target person), P = parent, T = teacher, H = head of school.

Age-specific content [27]	Wave 1 (2022/23)	Wave 2 (2023/24)	Wave 3 (2024/25)	Wave 4 (2025/26)	Wave 5 (2026/27)
**Civic literacy [9]**					
Sense of political effectiveness			S	S	
Political content in class			S	S	
Open classroom climate			S		
Participation in school			S		
Political and civil society participation	P		S		
Short scale of political interest (PIKS)			S		
Attitudes toward democracy			S, P		
Attitude toward a pluralistic society			S, P		
Hours: social studies / social sciences			H		
School-based support programs	P				
History of education	H	(H)	H, T	(H)	H
Understanding of politics			T		
Teaching quality in social science			T		
Teaching efficacy in social science			T		
Curricular content: social science			T		
**Well-being at school**					
Days absent		S			
Positive affect		S, P		S, P	S, P
School enjoyment		S		S	S

Concerns about school					S
Pressure to perform from parents		S		S	S
Pressure to perform in school		S		S	S
Bullying		S		S	S
School alienation [28]		S		S	S
Reading intentions and motives	S		S	S	S
**Vocational future**					
Career guidance through one's social network		S		S	S
Level of exploration with respect to career future		S, P		S, P	S
Clarity regarding career future		S		S	S
Applying for an apprenticeship					S
**Institution**					
Educational approach	H	H	H	H	H
Offer of a second foreign language	H	H	H	H	H
Teacher turnover	H	(H)	H	(H)	H
Institutional stress at school	H	(H)	H	(H)	H
Attitudes towards significance of performance	H	(H)	H	(H)	H
Social tensions			H		
Professional experience	H, T	(H), T	H, T	(H), T	H, T
Percentage of career changers and lateral entrants	H	(H)	H	(H)	H

Note. S = student (target person), P = parent, T = teacher, H = head of school, (H) = head of school who did not respond in the previous wave.

Sociodemographics [29]	Wave 1 (2022/23)	Wave 2 (2023/24)	Wave 3 (2024/25)	Wave 4 (2025/26)	Wave 5 (2026/27)
Gender identity	S, H	(H)	S, H	(H)	S, H
Sex at birth	S, H	(H)	S, H	(H)	S, H
Date of birth	S	S	S	S	S
Country of birth and date of arrival	S				
Nationality	S				S, P
Household composition/household size	S, P	S, P	S, P	P	S, P
Household income	P	P	P	P	P
Parents' country of birth	S				
Grandparents' country of birth	S				
Family relationship	S	S	S	S	S
Religion	S, P		S, P		S, P
Parental employment status	S, P	S, P	S, P	P	S, P
Parental occupations	S, P	P	S, P	P	P
Parental education	S				
Parental migration background	P	P	P	P	P
Material deprivation [30]	P	P	P	P	S, P
Childs‘ school history	S, P	P	P	P	P
Siblings	P		P		P

Note. S = student (target person), P = parent, T = teacher, H = head of school, (H) = head of school who did not respond in the previous wave.

References

1. Weinert, S., Artelt, C., Prenzel, M., et al. (2019). Development of competencies across the life course. In H. P. Blossfeld & H.-G. Roßbach (Eds.), *Education as a lifelong process* (pp. 57–81). Springer. https://doi.org/10.1007/978-3-658-23162-0_4

2. Gnambs, T., & Nusser, L. (2022). Out-of-level cognitive testing of children with special educational needs. *European Journal of Psychological Assessment*. https://doi.org/10.1027/1015-5759/a000736

3. Gehrer, K., Zimmermann, S., Artelt, C., & Weinert, S. (2013). NEPS framework for assessing reading competence and results from an adult pilot study. *Journal for Educational Research Online, 5*(2), 50–79.

4. Bremerich-Vos, A., Böhme, K., Weirich, S., & Köller, O. (2016). Kompetenzstufenmodelle im Fach Deutsch. In P. Stanat et al. (Eds.), *IQB-Bildungstrend 2016* (pp. 53–70). Waxmann.

5. Zimmermann, S., Gehrer, K., Artelt, C., & Weinert, S. (2012). *The assessment of reading speed in grade 5 and grade 9*. University of Bamberg. https://www.neps-data.de/Portals/0/NEPS/Datenzentrum/Forschungsdaten/SC4/1-0-0/com_rs_2012_en.pdf

6. Neumann, I., Duchhardt, C., Grüßing, M., Heinze, A., Knopp, E., & Ehmke, T. (2013). Modeling and assessing mathematical competence over the lifespan. *Journal for Educational Research Online, 5*(2), 80–109.

7. Hahn, I., Schöps, K., Rönnebeck, S., et al. (2013). Assessing scientific literacy over the lifespan: A description of the NEPS science framework and the test development. *Journal for Educational Research Online, 5*(2), 110–138.

8. Senkbeil, M., Ihme, J. M., & Wittwer, J. (2013). The test of technological and information literacy (TILT) in the national educational panel study: Development, empirical testing, and evidence for validity. *Journal for Educational Research Online, 5*(2), 139–161.

9. Alscher, P., Ludewig, U., & McElvany, N. (2022). Civic literacy: Zur Theorie und Messbarkeit eines Kompetenzmodells für die schulische politische Bildung. *Zeitschrift für Erziehungswissenschaft, 25*(5), 1221–1241. https://doi.org/10.1007/s11618-022-01085-0

10. Bäumer, T., Klieme, E., Kuger, S., et al. (2019). Education processes in life-course-specific learning environments. In H. P. Blossfeld & H.-G. Roßbach (Eds.), *Education as a lifelong process* (pp. 83–99). Springer. https://doi.org/10.1007/978-3-658-23162-0_5

11. Hawrot, A., Zink, C., & Nusser, L. (2026). *Instructional quality and teacher self-efficacy in NEPS starting cohort 8*. Leibniz Institute for Educational Trajectories. https://doi.org/10.5157/NEPS:SP130:1.0

12. Costa, J., Hawrot, A., & Stecher, L. (2022). *Active participation of children and young people in cultural activities*. Leibniz Institute for Educational Trajectories. https://doi.org/10.5157/NEPS:SP98:1.0

13. Stocké, V., Blossfeld, H. P., Hoenig, K., & Sixt, M. (2019). Social inequality and educational decisions in the life course. *Zeitschrift für Erziehungswissenschaft*. https://doi.org/10.1007/978-3-658-23162-0_6

14. Schulz, B., Horr, A., & Hoenig, K. (2017). *The position generator in the NEPS*. Leibniz Institute for Educational Trajectories. https://doi.org/10.5157/NEPS:SP23:1.0

15. Kristen, C., Edele, A., Kalter, F., et al. (2019). The education of migrants and their children across the life course. In H. P. Blossfeld & H.-G. Roßbach (Eds.), *Education as a lifelong process* (pp. 119–136). Springer. https://doi.org/10.1007/978-3-658-23162-0_7

16. Horr, A., De Paiva Lareiro, C., & Will, G. (2020). *Messung wahrgenommener ethnischer Diskriminierung im nationalen Bildungspanel (NEPS)*. Leibniz Institute for Educational Trajectories. https://doi.org/10.5157/NEPS:SP68:1.0

17. Gebel, M., & Heineck, G. (2019). Returns to education in the life course. In R. Becker (Ed.), *Research handbook on the sociology of education* (pp. 454–475). Edward Elgar. https://elgaronline.com/view/edcoll/9781788110419/9781788110419.xml

18. Bela, A., Feinauer, N., Gebel, M., Heineck, G., Kroh, J., & Mergard, K. (2018). *Returns to education across the life course in the national educational panel study*. Leibniz Institute for Educational Trajectories. https://doi.org/10.5157/NEPS:SP50:1.0

19. Bömmel, N., Kroh, J., Gebel, M., & Heineck, G. (2022). *Social inclusion as return to education in the national educational panel study (NEPS)*. Leibniz Institute for Educational Trajectories. https://doi.org/10.5157/NEPS:SP100:1.0

20. Kroh, J., Gebel, M., & Heineck, G. (2022). *Health returns to education across the life course*. Leibniz Institute for Educational Trajectories. https://doi.org/10.5157/NEPS:SP95:1.0

21. Wohlkinger, F., Blumenfelder, A. R., Bayer, M., von Maurice, J., Ditton, H., & Blossfeld, H. P. (2019). Measuring motivational concepts and personality aspects in the national educational panel study. In H. P. Blossfeld & H.-G. Roßbach (Eds.), *Education as a lifelong process* (pp. 155–169). Springer. https://doi.org/10.1007/978-3-658-23162-0_9

22. Hawrot, A., Wittmann, E., Steger, D., Schütz, A., & Gerlach, T. M. (2026). *Academic self-concept in the national educational panel study*. Leibniz Institute for Educational Trajectories. https://doi.org/10.5157/NEPS:SP132:1.0

23. Steger, D., & Schütz, A. (2026). Disambiguating tolerance of ambiguity: Introducing the MAAS-9 as a short, reliable, and multidimensional measure. *Journal of Personality Assessment*. https://doi.org/10.1080/00223891.2026.2645707

24. Steger, D., & Schütz, A. (2025). Assessing trust in science: Development and validation of a short scale. *Measurement Instruments for the Social Sciences, 7*, e16225. https://doi.org/10.5964/miss.16225

25. Taraszow, T., Gresch, C., & Kempert, S. (2024). The role of school context factors for gender role attitudes. *Youth & Society, 56*(5), 967–991. https://doi.org/10.1177/0044118X231205927

26. Goodman, R. (2001). Psychometric properties of the strengths and difficulties questionnaire. *Journal of the American Academy of Child & Adolescent Psychiatry, 40*(11), 1337–1345. https://doi.org/10.1097/00004583-200111000-00015

27. Fabian, P., Goy, M., Jarsinski, S., et al. (2019). Transition and development from lower secondary to upper secondary school. In H. P. Blossfeld & H.-G. Roßbach (Eds.), *Education as a lifelong process* (pp. 231–252). Springer. https://doi.org/10.1007/978-3-658-23162-0_13

28. Morinaj, J., Scharf, J., Grecu, A., Hadjar, A., Hascher, T., & Marcin, K. (2017). School alienation: A construct validation study. *FLR, 5*(2), 36–59. https://doi.org/10.14786/flr.v5i2.298

29. Schenck-Fontaine, A., Schönmoser, C., & Frembs, L. (2018). *Sociodemographic inequalities in education over the life course*. Leibniz Institute for Educational Trajectories.

30. Chincarini, E., Bayer, M., Kroh, J., Schenck-Fontaine, A., & Vogelbacher, M. (2022). *Ein erweitertes Konzept für die Messung von Armut im NEPS*. Leibniz Institute for Educational Trajectories. https://doi.org/10.5157/LIFBI:WP105:1.0
